# Supplementary figures and images for: A single amino acid polymorphism in a conserved effector of the multihost blast fungus pathogen expands host-target binding spectrum
Source: PLoS Pathog. 2021 Nov 10;17(11):e1009957. doi: 10.1371/journal.ppat.1009957 (PMC8608293; doi:10.1371/journal.ppat.1009957)

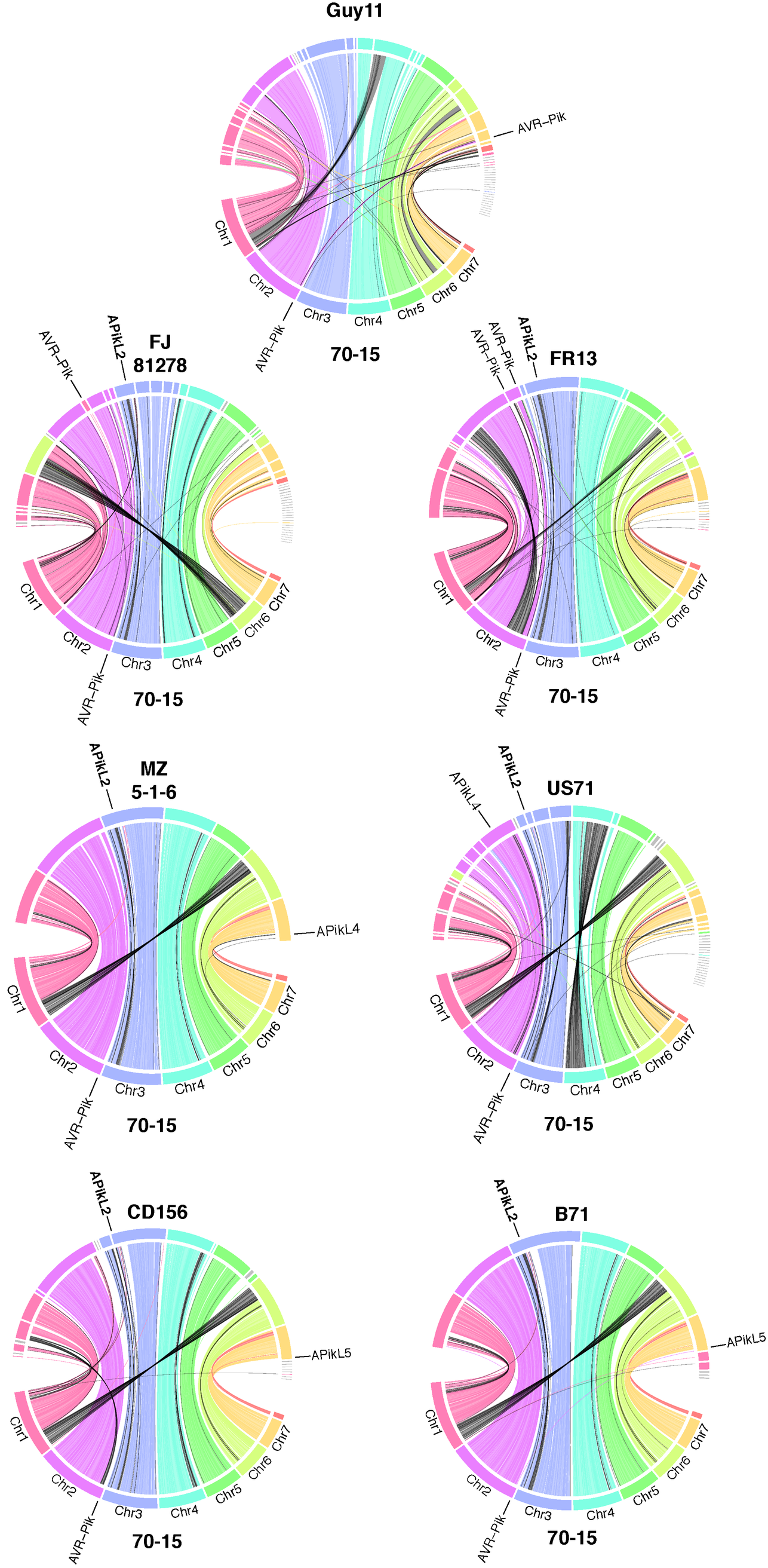

Supplement: S1 Fig — Whole genome alignments between the 70–15 reference genome assembly (EnsemblFungi assembly MG08) and seven isolates representative of five genetic lineages. Homologous chromosomes are color coded based on their best match in 70–15. Colored links between chromosomes show alignments >10 kb. Grey links show alignments in reverse direction (inversions). The location of each APikL gene is indicated. (TIF) [file ppat.1009957.s008.tif]

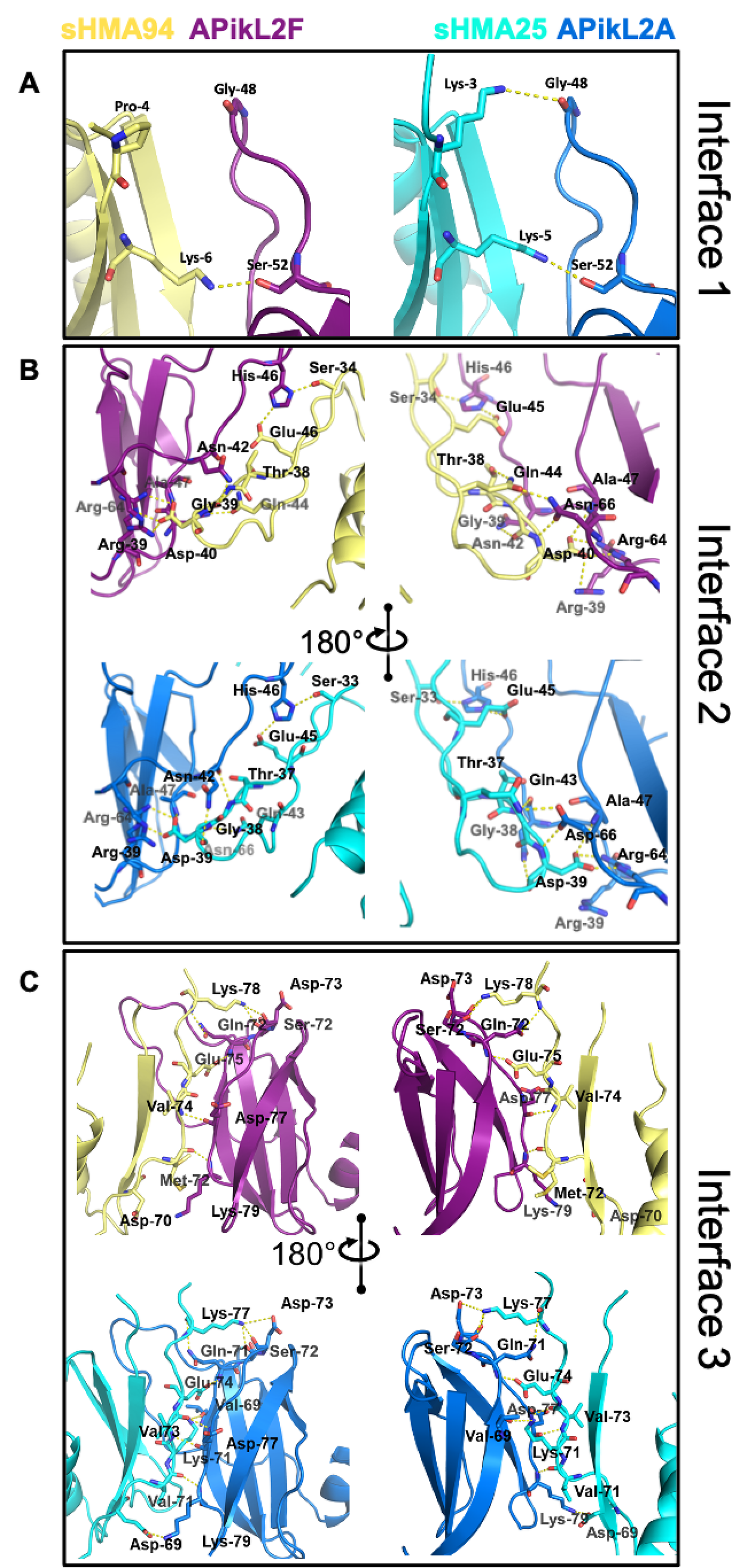

Supplement: S2 Fig — Cartoon representation of the two effector/sHMA complexes with ball and stick rendering of interacting residues as determined by PISA (EMBL-EBI). Hydrogen bonds are represented as a yellow dashed line with cut-off distance of 4 Å. A) Interface 1 B) Interface 2 C) Interface 3. (TIF) [file ppat.1009957.s009.tif]

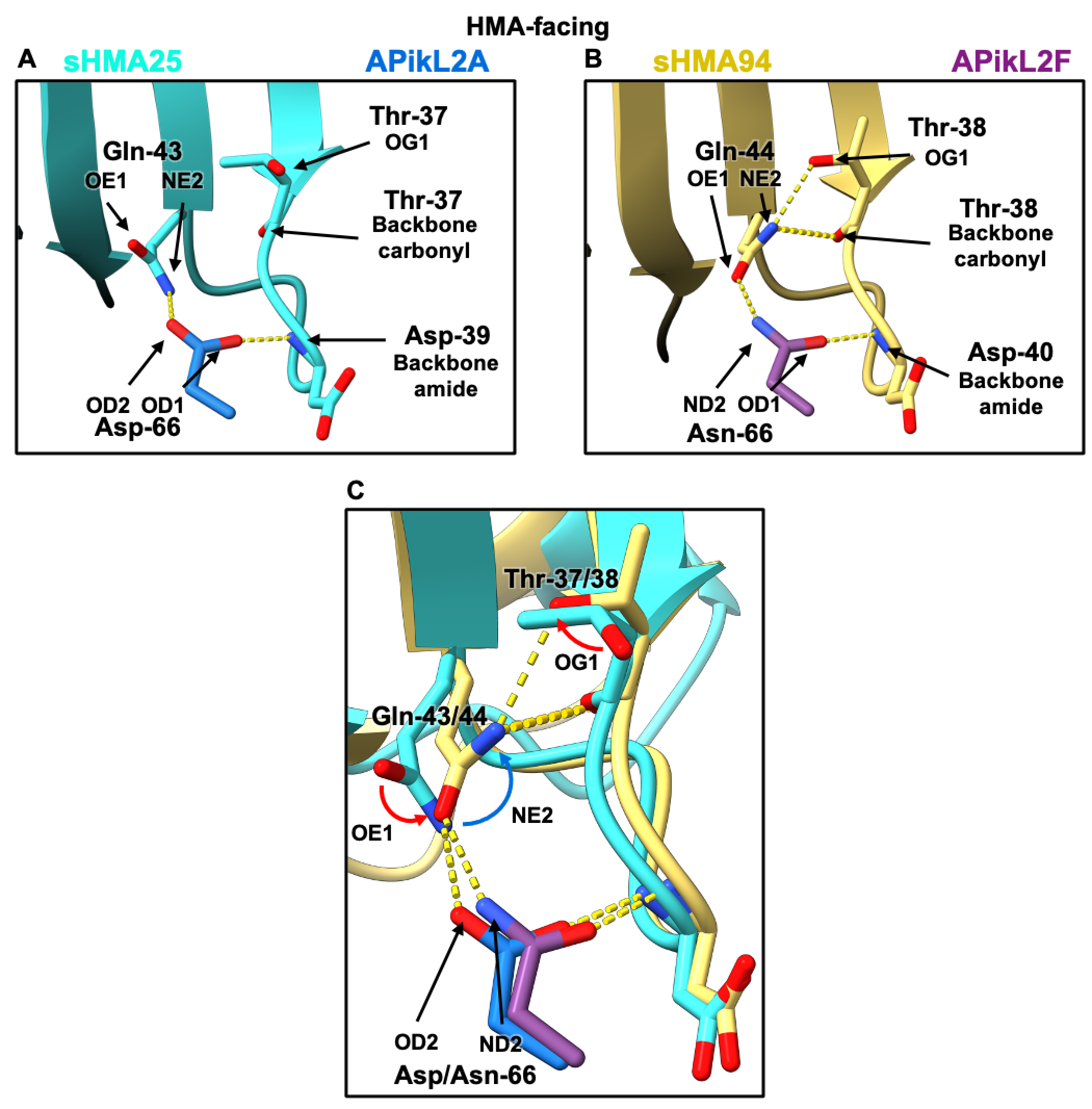

Supplement: S3 Fig — A, B) The hydrogen bonding networks of the APikL2A/sHMA25 and APikL2F/sHMA94 complexes around Gln-43/44, respectively. A) The OD1 atom of Asp-66 of APikL2 interacts with the NE2 atom of Gln-43 of sHMA25, preventing it from interacting with the OG1 atom and backbone carbonyl of Thr-37. B) The ND2 atom of Asn-66 interacts with the OE1 atom of Gln-44, allowing the NE2 atom of Gln-44 to interact with the OG1 atom and backbone carbonyl of Thr-38. C) Superimposition of the two APikL2/sHMA complexes. The blue and red arrows adjacent to Gln-43/44 indicate the movement of the NE2 and OE1 atoms between the two structures, exemplifying the rotation of the Gln-44 residue in the APikL2F/sHMA94 complex. Similarly, the red arrow adjacent to Thr-37/38 shows the movement of the OG1 atom in the APikL2F/sHMA94 complex, due to the interaction with the NE2 atom of Gln-44. (TIF) [file ppat.1009957.s010.tif]

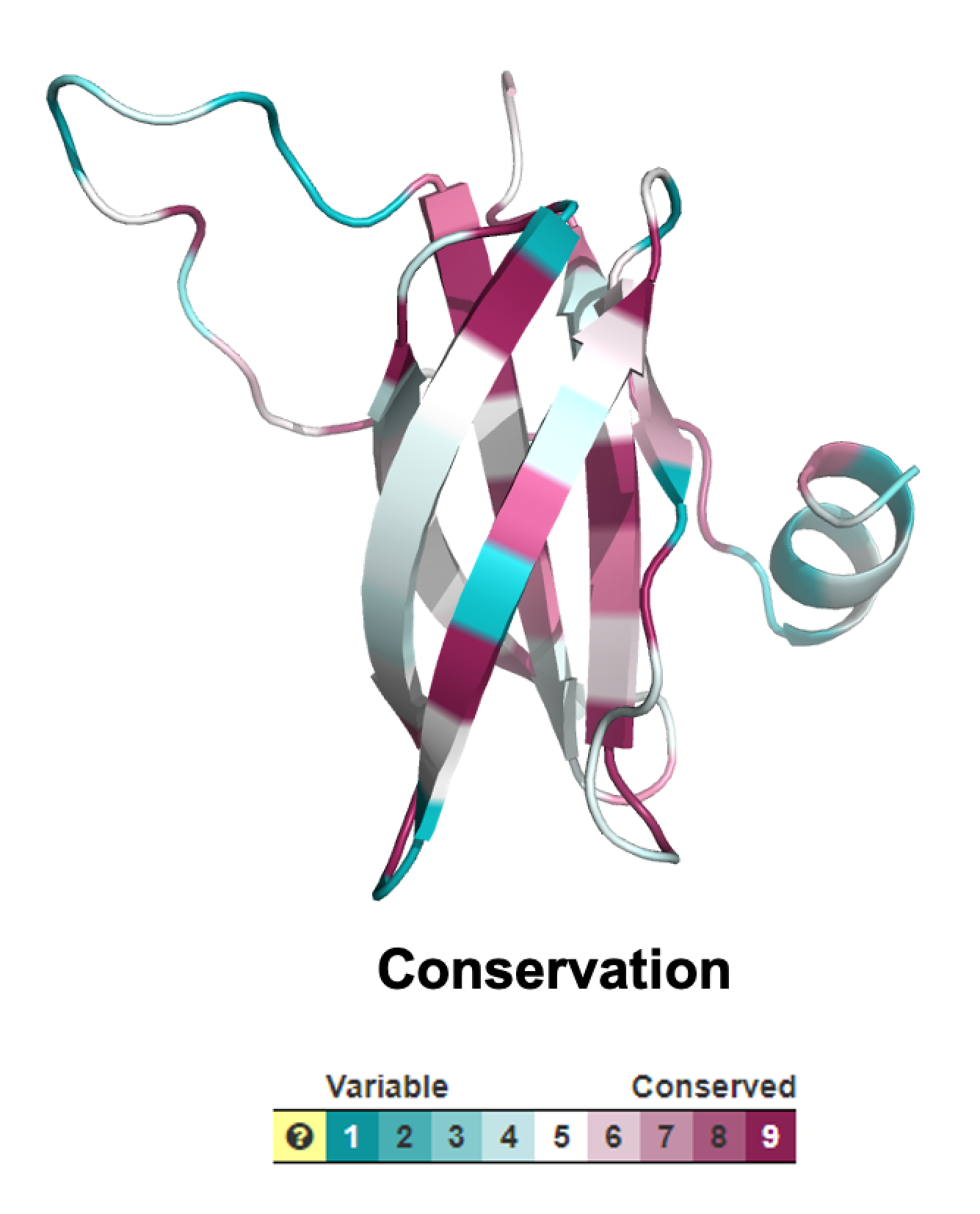

Supplement: S4 Fig — Conservation scores (determined by ConSurf) of amino acid residues in the APikL-family mapped to the crystal structure of APikL2. Colors indicate the conservation score and confidence interval. (TIF) [file ppat.1009957.s011.tif]

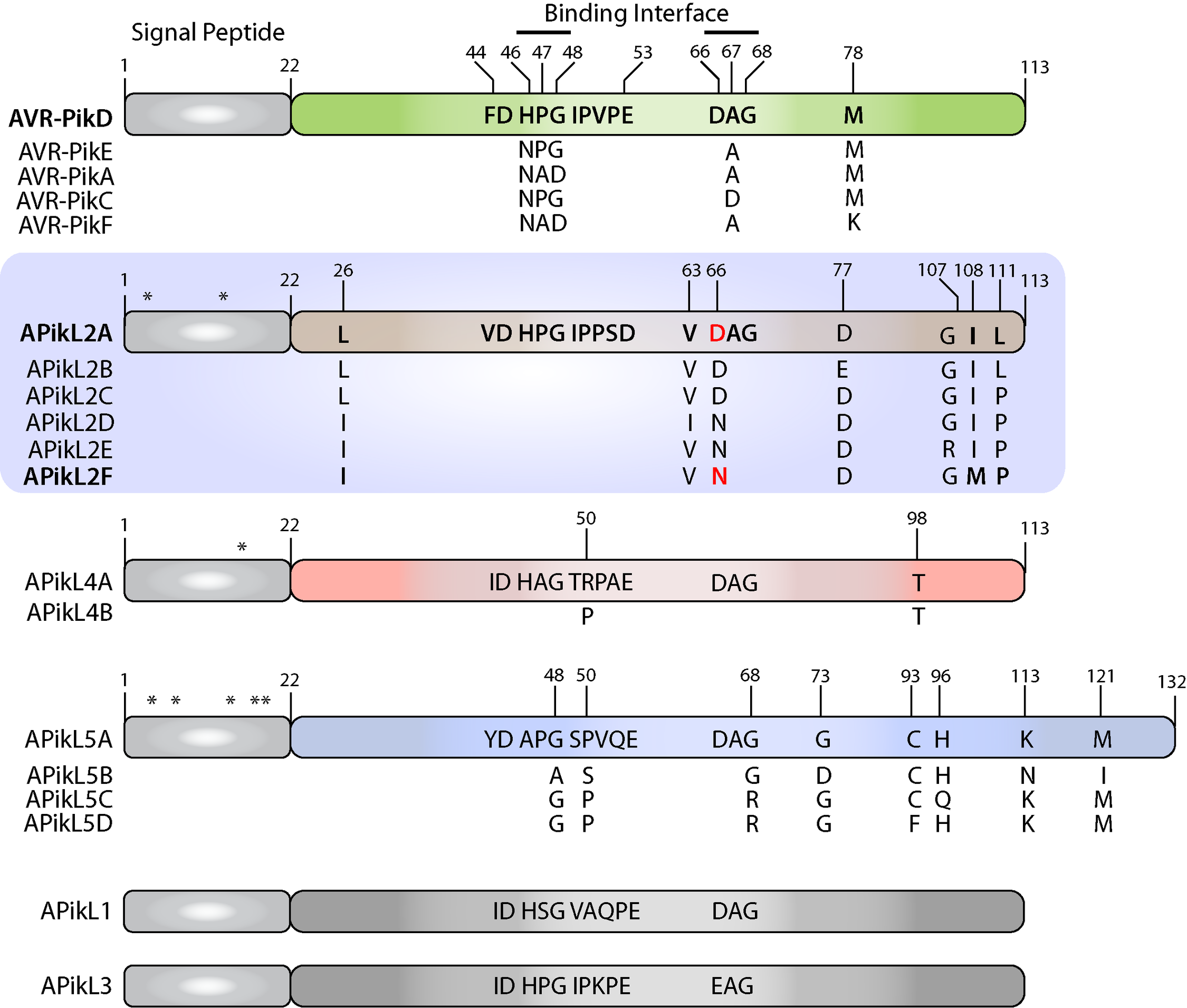

Supplement: S5 Fig — Schematic representation of the APikL family including all polymorphic sites. APikL effectors described in this study are highlighted in bold. The crucial polymorphism in APikL2 is highlighted in red. APikL family members in grey were not polymorphic in the population studied here. Asterisks indicate polymorphisms in the signal peptide. (TIF) [file ppat.1009957.s012.tif]

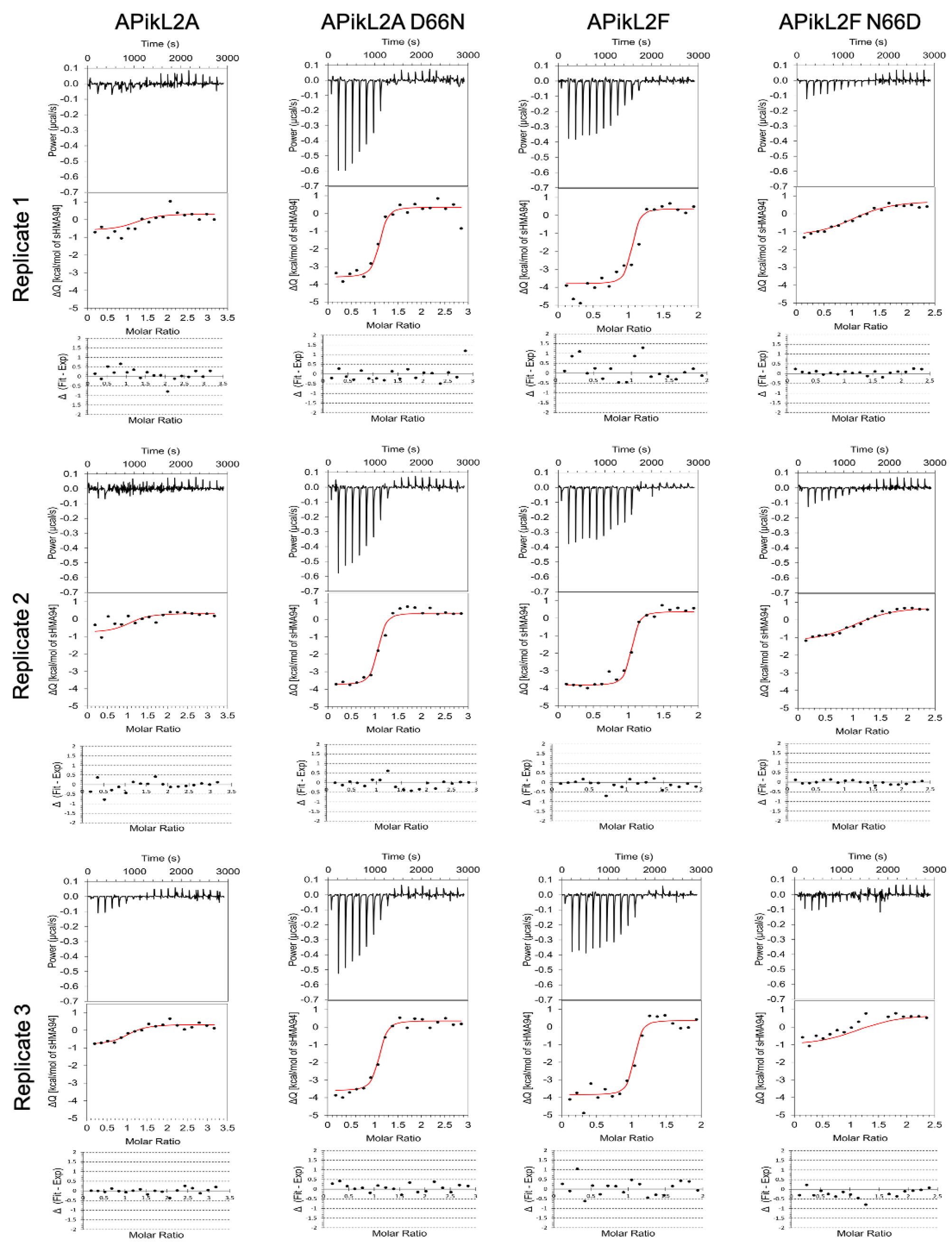

Supplement: S6 Fig — The top panels show heat differences upon injection of the effector into the cell containing sHMA94. The second panel shows integrated heats of injection (•) and the best fit (red line) to a single site binding model calculated using AFFINImeter ITC analysis software. The final panel shows the difference between the fit to a single site binding model and the experimental data; the closer to zero indicates stronger agreement between the data and the fit. (TIF) [file ppat.1009957.s013.tif]
