# Supplementary material for: A single amino acid polymorphism in a conserved effector of the multihost blast fungus pathogen expands host-target binding spectrum
Source: PLoS Pathog. 2021 Nov 10;17(11):e1009957. doi: 10.1371/journal.ppat.1009957 (PMC8608293; doi:10.1371/journal.ppat.1009957)
Supplement: Attachment — Submitted filename: Response_to_reviewers.docx [file ppat.1009957.s014.docx]

Response to editor

We thank the editor and reviewers very much for their thoughtful response. We have carefully addressed the reviewer comments and describe these in detail below.

Reviewer #1: While I enjoyed reading the manuscript, some issues should still be addressed:

Previous studies have demonstrated the functional relevance of Avr-Pik and other HMA-binding effectors in M. oryzae. I understand that the diversity of these effectors reflects a large redundancy in the effector repertoire of this pathogen. However, focusing on APikL2, what is the relevance of this effector in the outcome of pathogen infection? In the Discussion, the authors speculate that APikL effectors could play a role in speciation which would imply a significant role as virulence determinants. A deletion mutant would allow the authors to assess the relevance of the APikL2 gene in virulence and host specificity.

We are planning to initiate extensive genetics experiments involving both knockouts and allele complementation of the APikL genes in multiple blast fungus lineages. To be useful, these experiments would need to be quite comprehensive and involve multiple genes/alleles, lineages, and virulence assays on different host species. In addition, as pointed out by the reviewer, these experiments are complicated by functional redundancy (all members of the APikL family target HMA proteins) and would need to include multiple gene knockouts. In addition, single effector gene knockouts in *M. oryzae*, rarely yield measurable phenotypes. Thus, given the scale and resource investment required for these experiments we feel that they go beyond the current study, which is focused on the evolutionary structural analysis of the effector family. Adding in one or two knockouts without taking into account the broader context would dilute the focus of the paper and would probably push us to make premature potentially misleading conclusions.

The ancestral sequence reconstruction analysis is important to assess which mutation represent the recently derived one. However, the authors provide no details to the methods that they have applied for the ancestral sequence reconstructions. It is unclear if this was done using parsimony or maximum likelihood analyses? If it was conducted at the codon or amino acid level? Detailed information to the applied methods must be provided.

We thank the reviewer for this observation. We have amended the text and added this information in the “Material and Methods” section. “Ancestral state reconstruction was performed by a codon based maximum likelihood analysis with codeml (included in the PAML v4.9 package) according to [Yang et al 1997; Yang et al., 2007].”

Given the dataset, I am concerned about the application of the “site model” to identify sites under positive selection. If I understand correctly, the authors have included both paralogs and orthologs in their analyses. The site model assumes that all sequences evolve under the same regime, that is, selective pressure varies among sites and not among branches. In this case, I would recommend doing a branch-site model instead, using the candidate branch to define a foreground clade to test for sites under positive selection in this clade only.

We thank the reviewer very much for this useful feedback. Given the uncertainty about the underlying selection pressure on diverse APikL family members, we decided to remove the site model from our analysis. This does not change any of the conclusions in the paper.

There are several transcriptome studies of M. oryzae. Do the authors know if the APikL genes are expressed (also in the case of more than one gene)?

Unfortunately, we didn’t have the time or resources to perform a comprehensive gene expression analysis of the APikL genes across multiple lineage/host combinations. However, from limited public gene expression data containing APikL2 or other APikL members, we didn’t detect mRNA sequences matching APikL2. We have analysed two publicly available transcriptome datasets, including an early infection time course of the rice infecting isolate ina168 (containing AVR-Pik and APikL2) and field transcriptomics data from the wheat blast outbreak in Bangladesh (presumably containing APikL2 and APikL5). We re-mapped the raw reads of these datasets to the genomes of ina168 and B71 which is closely related to the BD wheat blast isolates. Although we did not detect transcripts for APikL2 in these datasets, we think it would be premature to draw major conclusions from this limited analysis as gene expression could be conditional and transient (e.g. tissue specific or host-lineage specific expression). Thus, we decided not to include these analyses in the final paper. However, for transparency, we provide these analyses as a Zenodo mini-paper (Langner et al., 2021) and added a statement in the text (discussion).

Reviewer #2: The genomic locations of the ApiKL family members don't seem to be discussed. Are these dispersed copies or do they occupy syntenic or idiomorphic locations among the genomes? I was not clear from the descriptions what was the scenario.

We thank the reviewer for the insightful comment, which prompted us to perform extensive analyses of APikL gene conservation and synteny across multiple lineages of M. oryzae. This analysis revealed that the APikL2 gene resides in a conserved and gene rich region on chr3 that contains genes that likely form a secondary metabolite gene cluster in all analysed isolates, whereas other APikL members locate to variable regions in the genome and inhabit gene poor locations without functional annotation. Overall, this analysis supports the view that genomic location might contribute to the broad conservation of APikL2. We added additional Figures and supplementary material as well as a new paragraph to the manuscript.

Part III – Minor Issues:

Reviewer #1: Page 3, upper paragraph: for readers outside the field of plant pathology, it would be good to define “biotrophic”.

We have added a short description of biotrophy to the text. “In biotrophic filamentous plant pathogens, i.e. pathogens that infect and proliferate in living host tissue, accelerated gene evolution is mostly documented at the level of coevolution with a single host species, but it has also been associated with host-range expansions and host jumps [1,14–21].”

Page 4: please write out terms for abbreviations “encoded by R or Pi genes”

We have included a better description of the terms R-gene or Pi genes in the text:

“Although *M. oryzae* effectors have evolved to modulate host processes to benefit the pathogen, they have been initially discovered through their AVR activities or the capacity to activate immune receptors (encoded by resistance (R) genes commonly called Pi genes) in resistant host genotypes.”

Page 7: How were the genes recognized as pseudogenes? Do these pseudogenes share some of the same mutations?

We have added a detailed description in the text. We have also identified an additional pseudogene in APikL4 in *Setaria* infecting isolates and corrected the text and Figure 1 accordingly.

“Ten of these genes appeared to be pseudogenized in isolates belonging to the *Oryza*, *Setaria* and *Brachiaria* lineage 2. We defined genes as pseudogenes when one of the following conditions were met: i) mutated or missing start codon, ii) truncated sequence in the assembly or iii) premature stop codons present. The identified pseudogenes included two AVR-Pik genes that were C-terminally truncated in rice infecting isolates, one APikL5 gene that is N-terminally truncated in the brachiaria infecting isolate Bd8401, four APikL4 genes in setaria infecting isolates in which the start codon was mutated, and three APikL2 genes with premature stop codons in rice infecting isolates from the US. The exact same mutations were shared between all APikL4 or APikL2 pseudogenes, respectively.”

Fig. 3A: It is interesting that ApikL5 also exhibits signatures of selection. Which sites are identified to be under selection in the ApikL5 branch? It would be interesting to provide this information in relation to the patterns in APikL2.

We decided to remove the site model analysis from the manuscript because we want to avoid making assumptions about the evolutionary pressure acting on diverse APikL family members. As correctly pointed out by the review, the site model assumes varying selective pressure on particular sites, but not branches. Even though overlapping genomic location, structural conservation of the HMA-binding interface, and conserved host targets of diverse APikL family members might indicate similar selective pressure across branches, we can’t make such an assumption with confidence. In addition, the inference of selection pressure on particular sites could be complicated by extreme dN/dS ratios and the lack of synonymous mutations in most terminal branches in the effector family.

Fig. 3B: The yellow dots are hardly visible. I recommend using another colour.

We changed color in Fig 3B to improve visibility.

Fig. 4A: It would be helpful to provide the amino acid positions on the top of the left schematic diagram.

We believe that the reviewer meant Figure 5A. We added the amino acid position of polymorphic residues between APikL2A and APikL2F to the schematic in Figure 5A. In the revised version of the manuscript, this is Figure 6A.

Reviewer #2: The classification of AVR-PikL members into familes is a little unclear to me from the methods and the results section. The TBLASTN approach took a query (AVR-Pik) and found homologs. But how do you know for strain B51 and moz-Z21 that the hits are AVR-PikL4 or AVR-PikL5? was a % identity cutoff used? was a tree used to classify the genes into their sub-families? Figure 1 shows the representation of the copies but I am not able to see how these were operationally assigned.

It seems like this is clearly shown in gene tree in Figure 2 - the tree confirms the assignments but I am not seeing if this gene tree information was used to make sub-family classifications?

We indeed used a percent identity cut-off to classify APikL family members. We included a short description in the “Material and Methods” section:

“APikL family members were classified using an amino acid percent identity cut-off of >90% for variants of the same effector.”

Is the Gene tree rooted? if you root it with a copy from a sister species -- I was going to look for this but I had trouble initially finding the ApiK gene named in databases, it would be useful if the Magnaporthe oryzae locus name was used somewhere in the paper so that links to databases like FungiDB could be directly accessed from this paper. Perhaps specifying the protein accession number and locus ID of the query ApiK protein in the Methods could be provided.

The effector gene encoding APikL2 is not present in the reference genome of strain 70-15. Only AVR-Pik has an accession number in the reference assembly MG08 (strain 70-15). However, APikL2 has an assigned gene ID in the wheat blast isolate B71 assembly (Peng et al., 2019). We added this information in the text:

“First, we used AVR-PikD (D allele of AVR-Pik; EnsemblFungi accession number: MGG_15972) as a query to identify sequence related proteins. This led to the identification of four effector candidates that we named APikL1, APikL2 (Syn. Pex75; accession number in isolate B71: BSY92_00423 according to [Peng et al. 2019]), APikL3, and APikL4.”

We further added protein and nucleotide fasta sequences of all sequences used in this study as supplementary material.

Figure 3- It is unclear why the dN axis needs to be as tall as dS - it would be easier to see the detail if truncated at 0.8? Not critical just a style suggestion.

We think it might be easier for readers that are not familiar with this type of analyses to interpret the dN/dS = 1 diagonal when both axes have the same length.

Please provide more detail in the methods for reproducibility of the computational analyses. Versions of programs (BUSCO, ASTRAL,RAxML) should be reported.

We have added version numbers to all programs used.

"Inference of a coalescence species tree by ASTRAL" -> please list which BUSCO marker set you used - sordariomycetes_odb10? ascomycota_odb10 ?

We have added database information “sordariomycetes_odb9” to the “Material and Methods” section.

Did the authors extract protein sequences of BUSCO markers - generally it doesn't return codiing sequences from BUSCO so I was not sure how they got nucleotide trees from the BUSCO analyses? Maybe the authors can elaborate.

In genome mode BUSCO generates both, amino acid and nucleotide sequences of identified genes. We extracted the nucleotide sequences of single copy BUSCO genes that were conserved in all isolates to generate the input ML trees for the ASTRAL tree. The corresponding function is described in the “Outputs” section of the BUSCO manual (https://busco.ezlab.org/busco_userguide.html#outputs).

Be consistent in tool name - TBLASTN is the tool name but I see it as tblastn and tBlastN in places.
The TBLASTN approach to generate gene models is appropriate but how was splicing confirmed? Usually tools like exonerate are better at getting splice boundaries and producing a coding sequence and proteins for downstream analysis. Was the protein generated from TBLASTN for the gene trees cleaned up in any way or just taking the translated sequence reported from TBLASTN?

We modified the text to make tool names consistent throughout.

The APikL effector genes do not contain introns. Protein sequences were extracted from TBLASTN and genomic sequences were extracted based on TBLASTN coordinates.

page 26 "effectors that included 10 pseudogenes." and page 27:
"To infer the APikL family tree we removed all pseudogene sequences from the analysis" - please detail how you identified pseudogenes?

We defined pseudogenes as all genes that lack a start codon, contain premature stop codons or have truncated N- or C-termini in the assemblies based on extracted genomic sequences. We added this information to the text:

“Ten of these genes appeared to be pseudogenized in isolates belonging to the *Oryza*, *Setaria* and *Brachiaria* lineage 2. We defined genes as pseudogenes when one of the following conditions were met: i) mutated or missing start codon, ii) truncated sequence in the assembly or iii) premature stop codons present. The identified pseudogenes included two AVR-Pik genes that were C-terminally truncated in rice infecting isolates, one APikL5 gene that is N-terminally truncated in the brachiaria infecting isolate Bd8401, four APikL4 genes in setaria infecting isolates in which the start codon was mutated, and three APikL2 genes with premature stop codons in rice infecting isolates from the US. The exact same mutations were shared between all APikL4 or APikL2 pseudogenes, respectively.”

Langner et al Zenodo 2021 - I don't see a DOI so was unable to verify what details of the data are deposited there (but I applaud the authors for making all supporting scripts and data available in a citeable archive)

We apologize for the delay in depositing this mini-paper in time. Due to the covid-19 situation, batch submissions of plasmids to Addgene were halted. Although this situation has not changed yet, we have received plasmid accession numbers and are preparing two Zenodo article submissions to support our manuscript, including sequences and accession numbers of all HMA-domain containing plasmids and APikL gene expression data described in this article (referred to as Langner et al., 2021a and b in the current manuscript version, respectively).

More detail could be added to the description of the APikL gene tree - "general topology of host species was inferred using NCBI" - do you mean this was a constraint? How was this topology integrated with the MEGA generated ML tree?

We removed this from the manuscript as it is not relevant for the final version. In figure 2, host-specific lineages were shown in taxonomic order (according to NCBI taxonomy). However, we decided to remove the tree that we retrieved from NCBI as it is not relevant for our analyses.

For the selection analyses (page 30) - I think the authors used the SITE model not the SIRE model - typo?

Ensembl not Ensemble (page 12, 13)

We have corrected several typos in the manuscript.

I addition to the reviewers comments, we received feedback in response to our bioRxiv prepublication. We have taken these comments into consideration and have addressed the following issues in the revised version of the manuscript.

In page 6, you indicate "First, we used Avr-Pik as a query". I would suggest specifying that it's Avr-PikD.

We amended the text accordingly. Additionally, we have added gene accession numbers for AVR-Pik (EnsemblFungi assembly MG08, isolate 70-15) and APikL2 (Genbank assembly MQUD00000000.1; isolate B71) to the manuscript.

In page 8, you indicate 2 variants for APikL4 and 5 for Avr-Pik, but the Figure 2 seems to show 4 and 11, respectively?!

As we will come back to APikL2A and APikL2F quite often, I would also suggest to write, on Fig 2, where are APikL2A, B, C, D, E and F.

The offset in the branches leading to AVR-Pik, APikL4 and 5 was the result of how the phylogenetics software plotted the tree. We have corrected this in the final figure and added the variant ID to APikL2 branches for clarity.

In page 12, you indicate that 4 AA are polymorphic. The alignment is not shown and I didn't find any data on the manuscript about the DNA or protein sequences for the APikL candidates. You should consider providing at least:

- APikL2A and APikL2F that you used for Y2H test

- APikL4 and APikL5 that you used for prediction modelling

And ideally, all the sequences from Fig 2

We have included all amino acid and nucleotide sequences as supplementary material.

In page 12, I would specify that it is from 16 possible combinations, that you tested 12 variants.

We have added this to the text.

In page 18, it is important to remember that the D66N variants (APikL2D/E/F) are not detected in lineage infecting Setaria (as noted in the discussion). Therefore, while the D66N mutation can expand the binding spectrum to sHMA94-like targets, there is however no possible co-evolutionary scenario between D66N and the Setaria sHMA94, also the sentence should be rephrased.

Also in page 20, you shouldn't write "expands the effectors binding spectrum to the new target, sHMA94."

We have changed the sentence on page 19 to “sHMA94-like HMA domains” to better reflect the likely evolutionary scenario. We did not change the paragraph on page 21 as this is merely a summary of our observations and does not contain interpretation of the results.

In FigS4, which lists all polymorphic sites, the polymorphism in position 107 is not indicated for APikL2, while it is described in Fig 8B.

In FigS4, the meaning of the * is not given. Is it polymorphism in the signal peptide?

We have added the polymorphism at position 107 to the figure and amended the figure legend to explain the asterisks which indeed show polymorphisms in the signal peptide.
